# Patellar Tendon Z-plasty Approach for Comminuted Tibial Plateau Fractures

**DOI:** 10.7759/cureus.97190

**Published:** 2025-11-18

**Authors:** Pavlos Mouratidis, Georgios E Prelorentzos, Aristeidis Koutsopoulos, Theodoros Vatistas, Georgios Sarantopoulos, Dimitrios Polyzois, Demetris Constantinou, Charis Papadaki, Ioanna Aggelaki, Panagiotis Tsailas

**Affiliations:** 1 4th Department of Orthopaedics, KAT Attica General Hospital, Athens, GRC; 2 Department of Orthopaedics, University of Ioannina, Ioannina, GRC

**Keywords:** comminuted, open reduction internal fixation, patellar tendon, plateau fracture, z-plasty

## Abstract

Introduction

This study aims to describe the surgical technique and evaluate the functional and radiological outcomes of comminuted tibial plateau fractures treated using the patellar tendon Z-plasty approach.

Materials and methods

A retrograde analysis was conducted with clinical and radiological data on nine patients from 2011 to 2025 with an average age of 42 years (range, 20-70 years) who were admitted for tibial plateau fractures with significant comminution involving the intercondylar area and/or the posterior parts of the condyles. Surgery was performed using a midline incision with patellar tendon Z-plasty. This technique provides full visibility to the proximal tibia and makes anatomic reduction easier under direct vision.

Results

Patients were followed up for an average of 5.3 years (range, 0.5-11 years). No problems with the extensor mechanism were encountered. The range of motion at the final follow-up was normal (0^o^-140^o^), with only one patient developing a fixed flexion contraction of 8^o^ due to severe posttraumatic osteoarthritis. Assessment of the knee joint function at the final follow-up was conducted using the Knee Injury and Osteoarthritis Outcome Score (KOOS).

Conclusion

Patellar tendon Z-plasty is a safe approach that can be used in comminuted plateau fractures. It makes anatomic reduction easier under direct vision, through a single anterior incision, while having excellent postoperative results.

## Introduction

Comminuted tibial plateau fractures expose the surgeon to a vast spectrum of articular, metaphyseal, and soft-tissue injury patterns, and no single exposure can address every possible combination [1]. The three-column fixation concept described by Luo et al. has been a valuable planning tool based on the CT image of the fracture [2]. Three-dimensional (3D) CT mapping studies have confirmed that up to 40% of bicondylar injuries harbor distinct posteromedial or posterolateral fragments and that the correct incision choice can be challenging [1-3].

Patellar tendon Z-plasty is not a new concept. It was first described by Schatzker in 1968 for post-traumatic contracture of the knee [4]. Using this approach, a single midline incision can address both condyles, especially with severe comminution and with posterior involvement, which would require multiple incisions, and possibly intraoperative changes to the positioning of the patient to address the posterior fractures, as well as fractures of the intercondylar area.

## Materials and methods

This was a retrograde analysis (non-consecutive case series) conducted at KAT Attica General Hospital, Athens, Greece, of clinical and radiological data for adult patients (>18 years old) with tibial plateau fractures with significant comminution involving both the lateral and the medial plateau, with involvement of the posterior parts of the condyles and/or the intercondylar area, treated with patellar tendon z-plasty approach and open reduction internal fixation from 2011 to 2025. Patients younger than 18 years old, with plateau fractures type I-III, with no posterior part comminution, were excluded from the study. The study was approved by the Scientific Committee, KAT Attica General Hospital (approval number: 24788/10-10-2025), and informed consent was obtained from all participants.

Study population

Nine patients were found to match the inclusion criteria. Tibial plateau fractures were classified using the Schatzker and three-column classification systems. Of the nine fractures analyzed, two were type IV fractures, three were type V, and four were type VI fractures per Schatzker. Based on Luo’s three-column classification, two fractures were two-column with medial and lateral involvement, and seven were three-column fractures. All fractures were fixed with two plates, K-wires, and free screws, except for one, in which only one plate was used. In the patient in whom only one plate was used, the comminution was medial and posterior with no lateral involvement. 

Surgical technique

**Figure 1:**
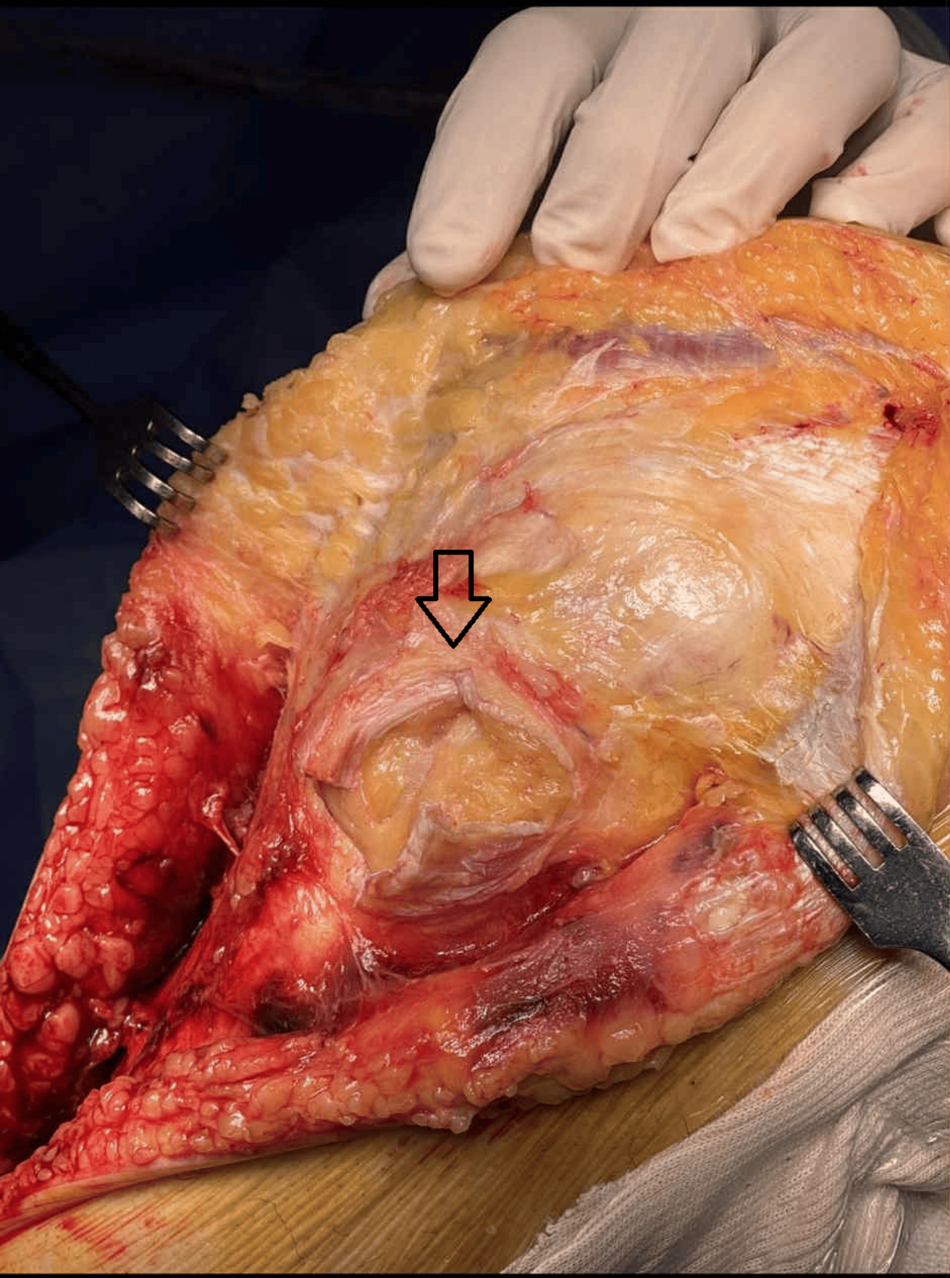
Patellar tendon Z-plasty A perpendicular incision in the middle of the patellar tendon, a transverse cut  proximally and laterally and another transverse cut  distally and medially

**Figure 2:**
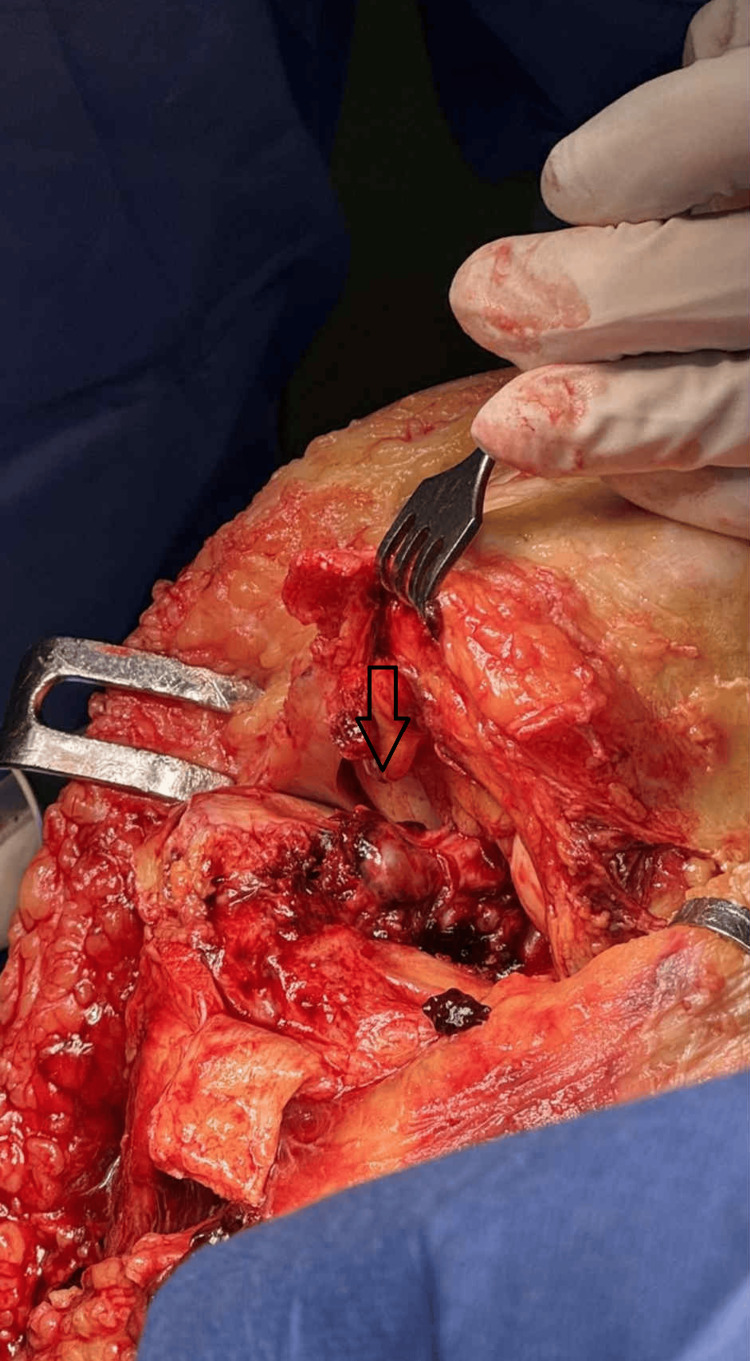
Intraarticular view Patellar Tendon Z-plasty offers a direct view of the articular surface that aids fracture reduction

Surgery was performed with the patient supine on a radiolucent flat-top operating table and the lower extremity in neutral position. A quadrilateral frame was used in order to keep the knee flexed and the foot in the air. An extended straight midline incision was made starting 5-8 cm proximal to the superior pole of the patella to as far as needed distally to address the tibial fracture. Full-thickness flaps were raised. A perpendicular incision was first made in the middle of the patellar tendon, a transverse cut was then made proximally and laterally, and another transverse cut was made distally and medially, leaving about 1 cm of tendon stump for suture placement during the final repair. Once the two halves of the tendon were lifted from the underlying tissues, the capsule and synovium were cut transversely below the menisci. Medially, the transverse cut extended to one-third of the anterior plateau, so as to avoid cutting the medial collateral ligament. Laterally, the transverse cut extended to the posterolateral corner of the plateau, just as with the standard lateral approach. The capsule, the attached menisci, and the patella were then lifted up, exposing the whole proximal tibia (Figures 1, 2)

This approach provides an excellent view of the articular surface of the tibia, even of the posterior part. Anatomic reduction of the articular surface is achieved under direct vision. Fragments are temporarily held together with K-wires. On the lateral side, the tibialis anterior was raised from its origin to allow placement of a lateral plate. On the medial side, an anteromedial plate was placed over the periosteum.

**Figure 3:**
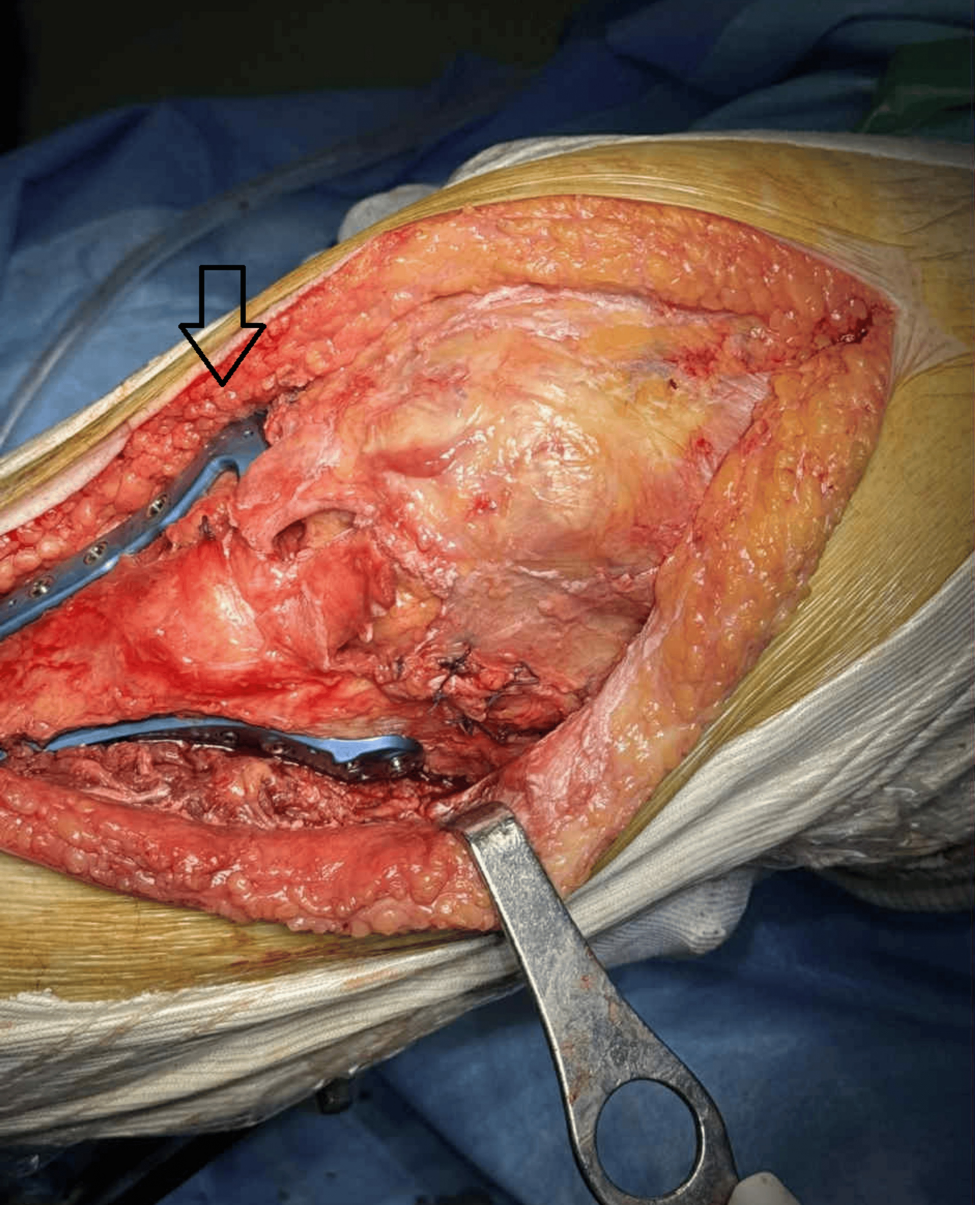
Open reduction internal fixation Placement of two plates is performed

**Figure 4:**
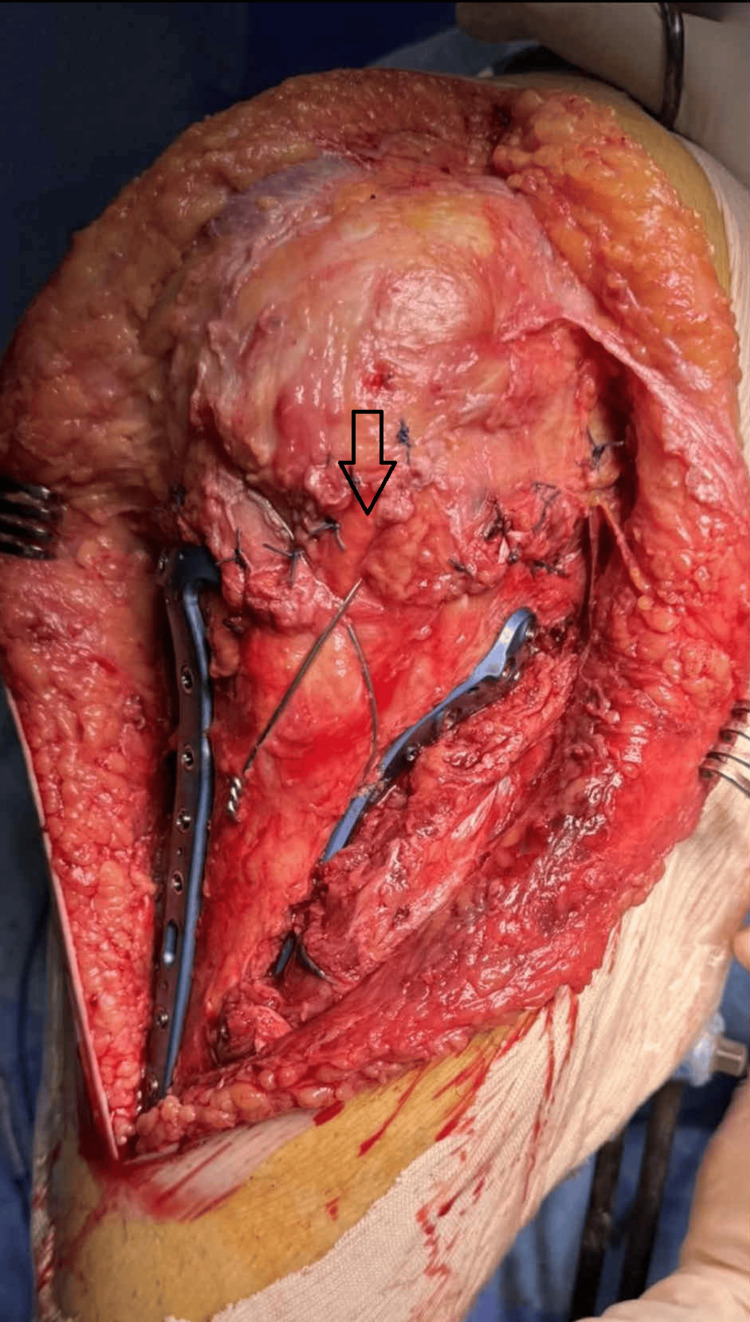
Prophylactic wire placement Capsule closure and prophylactic figure-of-8 wire placement

Fracture in the intercondylar area affecting the anterior cruciate ligament may be addressed with heavy sutures or thin wires. Occasionally, the lateral meniscus may be torn and found inside the fracture; in that case, it was sutured back to the capsule using No. 2 non-absorbable sutures. The limbs of the Z-plasty were secured with interrupted No. 2 non-absorbable sutures, starting with the transverse ends and then the midline incision. The transversely cut capsule was repaired, and finally, a prophylactic figure-of-8 wire was placed around the proximal part of the patella and through a transverse drill hole distal to the tibial tubercle, to protect the repair of the patellar tendon. The tension of the prophylactic wire should be so that at 90 degrees of flexion, the tendon should feel just lax (Figures 3, 4).

Postoperative management

**Figure 5:**
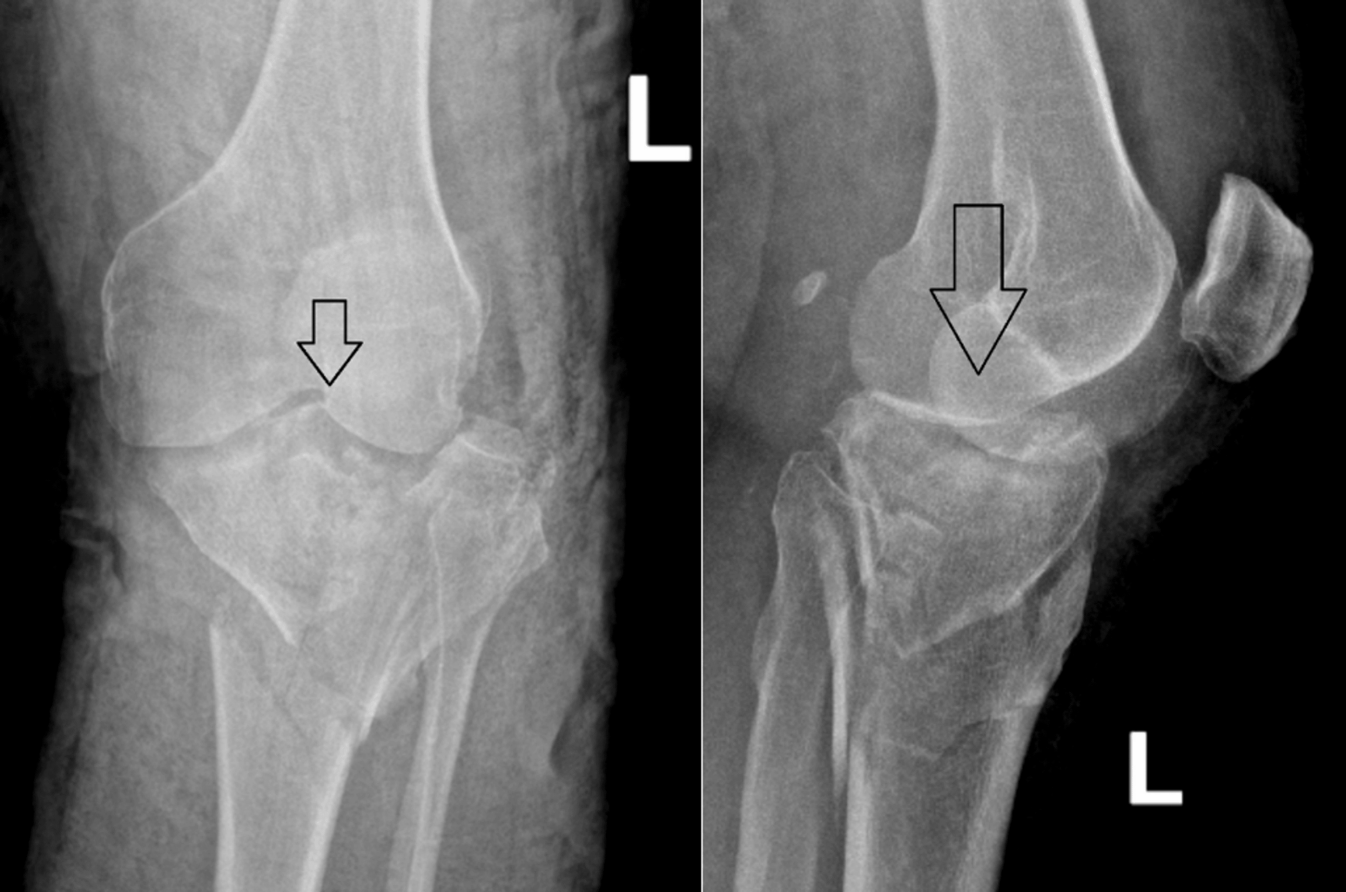
Preoperative X-ray Plateau fracture type V per Schatzker

**Figure 6:**
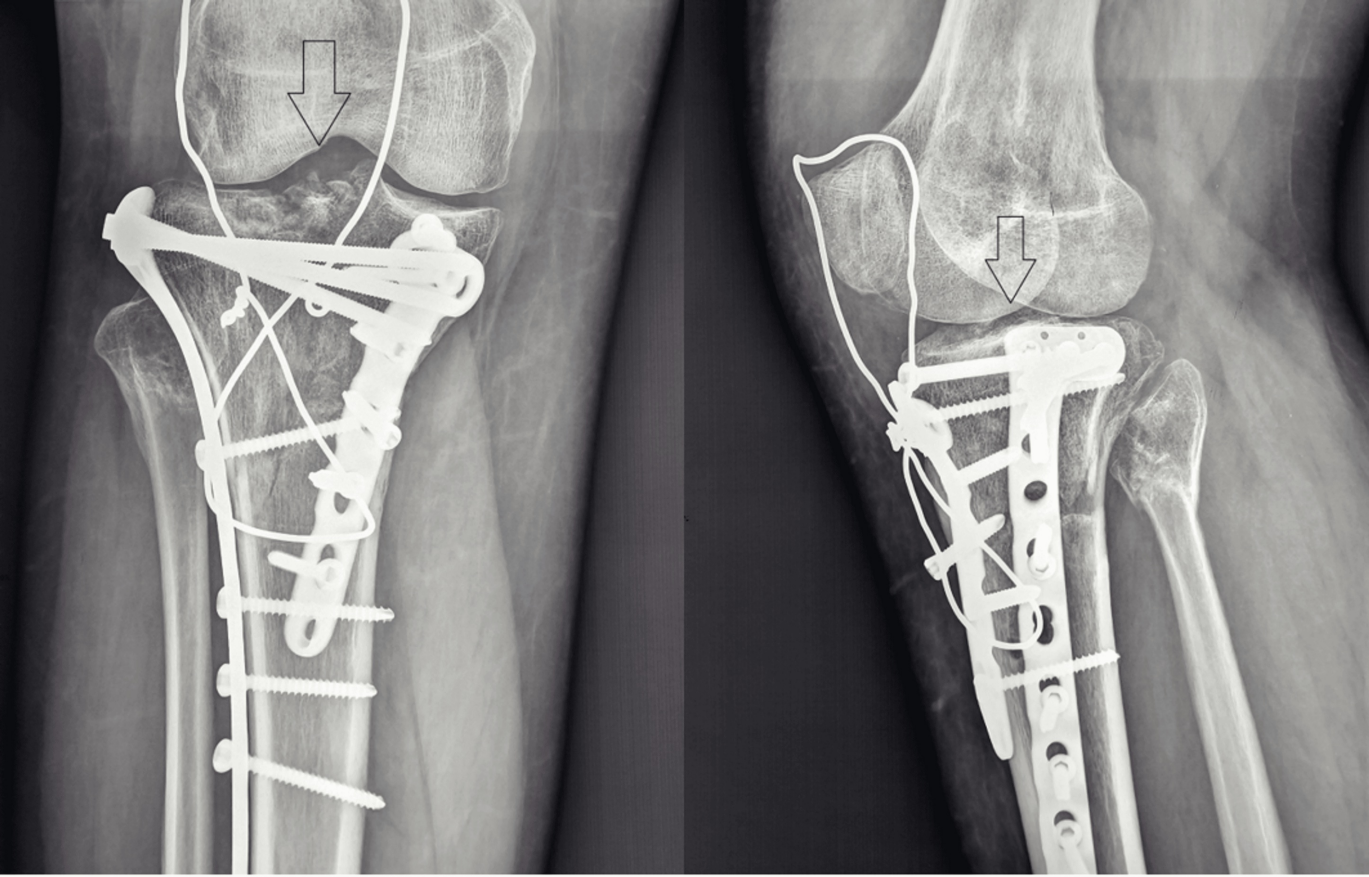
Postoperative X-ray at the one-year follow-up

Antibiotics were administered for 48 hours, and antithrombotic prophylaxis for six weeks. A hinged knee brace was used to control the range of motion (ROM), starting with 0^o^-40^o^ for two weeks, and with an aim to reach 90° at six weeks postoperatively. We believe that initiating an early ROM is essential for achieving optimal postoperative outcomes. The patient was allowed to do isometric and isokinetic exercises. For the first six weeks, no weight bearing was permitted. After six weeks postoperatively, the patients were allowed to perform knee flexion beyond 90^o^, while gradually increasing weight bearing was allowed. Full weight bearing was usually achieved by three months. Patient with a plateau fracture type 5 preoperatively and at one-year follow-up (Figures 5, 6).

Measurement of clinical outcomes

The Knee Injury and Osteoarthritis Outcome Score (KOOS) was utilized to evaluate the clinical outcome, including pain, symptoms, activity of daily living (ADL), sport and recreational activities, and knee-related quality of life (QOL). The KOOS, a validated knee-specific instrument, is commonly used to evaluate the progression of functional recovery and quality of life (QOL) following tibial plateau fractures [5]. A normalized score (100 indicating no symptoms and 0 indicating extreme symptoms) was calculated for each subscale. A summarized scale of the KOOS score cannot be calculated due to the heterogeneity of the subscales. 

## Results

A total of nine patients were included with an average age of 42 years (range, 20 to 70), comprising six men and three women. Follow-ups were done by the treating surgeon through clinical and radiographic evaluation. In our series, there was no case of postoperative infection; all patients proceeded to full fracture union, there were no complications regarding the Z-plasty of the patellar tendon, and all patients achieved full ROM (0-140°) by week 12 and recovered complete functionality by week 24. One of the patients with severe comminution of the articular surfaces finally developed postoperative arthritis and, in the latest follow-up, had a fixed flexion contracture of 8°. Despite the arthritis, the patient is still functional and does not seek further treatment. The mean follow-up was 5.8 years. Cerclage wire removal was not routinely performed unless it caused soft tissue irritation (three patients). Despite the severity of the fractures addressed with this technique, all the patients returned to their normal lives and remained satisfied. Table 1 gives the characteristics and outcomes of the patients.

**Table 1:** Patients characteristics and results ORIF: open reduction internal fixation; ROM: range of motion; M: male; F: female

Patient number	Sex	Age (years)	Follow-up Duration (years)	Schatzker Classification type	Three-Column Classification type	ORIF Plates	ROM	Wire Removal	Wire Breakage
1	M	39	11	V	Three Column	Two Plates	0-135	Yes	No
2	M	23	13	V	Three Column	Two Plates	0-140	No	No
3	F	70	1	V	Three Column	Two Plates	0-130	No	No
4	M	67	1	VI	Three Column	Two Plates	0-140	No	No
5	F	39	4	IV	Two Column Medial Posterior	Two Plates	0-140	-	Yes
6	M	32	1	VI	Three Column	Two Plates	0-130	No	No
7	M	26	9	VI	Three Column	Two Plates	8-100	Yes	No
8	F	20	10	IV	Two Column Medial Posterior	One Plate	0-140	Yes	No
9	M	60	0.5	VI	Three Column	Two Plates	0-130	No	No

Median KOOS scores for the five subscales were 91.9 (interquartile range (IQR), 71.5-100) for ‘symptoms’, 89.7 (IQR 80-100) for ‘pain’, 94.1 (IQR 84-100) for ‘ADL’, 46.7(IQR 0-100) for ‘sports and recreation’, and 62.5 (IQR 37,5-100) for ‘knee related QoL’ (Table 2). The lowest score was observed in sports activities, including running, jumping, and deep squats. Patients reported that pain and function continued to improve even one year after the surgery.

**Table 2:** Functional outcomes based on KOOS score KOOS: Knee injury and Osteoarthritis Outcome Score; QoL: quality of life

Patient number	Pain score	Symptoms score	Activities scores	Sports and Recreation score	QoL score
1	100	100	100	100	100
2	96	96.5	95.6	95	93.75
3	80	96.5	84	20	37.5
4	80.6	89,.3	89,.7	20	54
5	82	85.6	95.4	35	55
6	83.4	100	92.6	0	43.7
7	97	71.5	100	35	37.5
8	97	96.5	95.6	95	87.5
9	91.7	89.3	94.1	20	50
MEAN	89.7	91.9	94.1	46.7	62.1

## Discussion

Patellar tendon Z-plasty is a safe technique that can be very useful for anatomic reduction of bicondylar tibial plateau fractures that have posterior comminution. Under direct view of the articular surface, the surgeon can ensure the condylar width is restored and that there is no articular step off. We usually use this approach for Schatzker type V and VI fractures [6] with posterior fragments or intercondylar comminution. To our knowledge, this is the first case series describing long-term results of this technique.

In this study, no failures of the repair of the patellar tendon were observed. After healing of the patellar tendon, all patients had an excellent ROM comparable to the healthy knee. Only one patient with a severely comminuted Schatzker type V fracture had 8^o^ of fixed flexion, which, however, has not limited his daily activity.

The findings of Kumar and Whittle [7] and Marsh et al. [8] identify an association of imprecise articular reduction with lower functional outcomes. Evidence suggests that indirect articular reduction, based solely on radiographic assistance, can yield variable and sometimes suboptimal results [9]. Patellar tendon Z-plasty offers direct vision to the plateau, so a more accurate anatomic reduction is possible. Additionally, we minimize the use of fluoroscopy during surgery. Many studies have debated the use of a single or double incision for double plating and its associated complications. Bicondylar plateau fractures are high-energy fractures and often have high rates of soft tissue complications such as swelling, blister formation, abrasions, and open fractures, even compartment syndrome [10]. Extensive dissection through the already compromised soft-tissue envelope using a single midline incision is thought to disrupt the vascularity of the skin flaps, leading to soft tissue problems and infection [11]. However, Guild et al., in a study with 346 patients, postulated that both single and double incision approaches have comparative safety and that, even with the use of double incisions, if not spaced correctly, skin problems may arise [12].

Our goal was to describe the Z-plasty approach as an effective approach that can be used for certain plateau fracture types, not to compare it with the traditional approaches. We did not encounter any problems with wound healing or infection. The wound dressing change was performed at our clinic twice a week. This approach also preserves the option for total knee arthroplasty in case of posttraumatic osteoarthritis using the same anterior midline incision. With this technique, if a posterior buttress plate is required, a second posteromedial approach may still be performed, with the prerequisite of an adequate skin bridge between the two incisions. In our study, we used an extra posteromedial approach in two patients. Placing a posterior buttress plate is easier because the reduction is under direct vision of the articular surface. In case of skin necrosis or infection, external fixation and flap coverage might be needed [13]. The medial gastrocnemius muscle flap has been used for soft tissue defects and complications around the knee with excellent results [14].

Z-plasty has been used for lengthening or shortening the patellar tendon. Indications include post-traumatic scarring after tibial-tubercle and plateau fractures. This technique has also been described in the management of patella baja after total knee arthroplasty and high tibial osteotomy, with excellent outcomes at restoring patella height and with no reruptures [15,16]. In these studies, however, a modified technique with four limbs was used. Patellar tendon shortening was described for patients with cerebral palsy and flexed knee gait [17]. A similar, in concept, approach to the knee in a trauma setting includes tibial tubercle osteotomy (TTO) [18,19]. TTO has been described for comminuted and displaced plateau fractures and provides adequate exposure to the proximal tibia. However, it has been associated with serious postoperative complications, including fracture, nonunion, and skin necrosis [20,21]. We believe that creating one more fracture at an already compromised site would put the bone healing and the fixation at risk.

Limitations

To our knowledge, this is the first study to analyze the surgical technique and long-term follow-up of patellar tendon Z-plasty for plateau fractures. However, this study was a case series with only a small number of patients, so its results cannot be generalized. There was no control group to compare our results with. It was a retrospective analysis, and this could impact the study’s conclusions. Further research, such as randomized control trials, prospective control trials, and comparative effectiveness trials, is needed to confirm the results of this study and to address its limitations. The surgery and follow-up were conducted by the treating surgeon, which may have introduced observer bias.

## Conclusions

Patellar tendon Z-plasty is a valuable approach to plateau fractures with posterior comminution, providing a direct view to the articular surface of the tibia and thus enabling the anatomic reduction of the fracture fragments. 
